# Supplementary material for: Evolution and diversity of biomineralized columnar architecture in early Cambrian phosphatic-shelled brachiopods
Source: eLife. 2024 Apr 10;12:RP88855. doi: 10.7554/eLife.88855 (PMC11006422; doi:10.7554/eLife.88855)
Supplement: Supplementary file 1. [file elife-88855-supp1.docx]

**Supplementary file 1**. Average dimensions and ratios of ventral and dorsal valves of *Latusobolus xiaoyangbaensis* gen. et sp. nov. from the Cambrian Series 2 Shuijingtuo Formation, South China.

| V |  | L | W | | H | | L_m_ | L_ms_ | | W_ms_ | | L_n_ | L_p_ | | W_p_ | | L_pl_ | W_pl_ | | L_g_ | W_g_ | | L_p-i_ | | W_p-i_ | L_p-e_ | | W_p-e_ | | A | A_g_ | | Pi | Pu | | L/W | | H/L | |
| --- | --- | --- | --- | --- | --- | --- | --- | --- | --- | --- | --- | --- | --- | --- | --- | --- | --- | --- | --- | --- | --- | --- | --- | --- | --- | --- | --- | --- | --- | --- | --- | --- | --- | --- | --- | --- | --- | --- | --- |
| N |  | 17 | 15 | | 20 | | 11 | 19 | | 19 | | 5 | 14 | | 13 | | 6 | 5 | | 17 | 20 | | 12 | | 11 | 12 | | 11 | | 19 | 14 | | 28 | 40 | | 14 | | 17 | |
| Mean |  | 1244 | 1237 | | 239 | | 655 | 223 | | 275 | | 523 | 165 | | 615 | | 321 | 634 | | 112 | 120 | | 157 | | 29 | 277 | | 51 | | 129° | 34° | | 0.5 | 6.5 | | 95.17% | | 18.02% | |
| Min |  | 775 | 806 | | 111 | | 418 | 169 | | 206 | | 326 | 70 | | 320 | | 168 | 456 | | 42 | 85 | | 46 | | 8 | 105 | | 9 | | 117° | 24° | | 0.3 | 2.3 | | 90.43% | | 11.58% | |
| Max |  | 2325 | 2417 | | 519 | | 1460 | 267 | | 327 | | 759 | 316 | | 929 | | 434 | 857 | | 201 | 172 | | 303 | | 55 | 516 | | 122 | | 142° | 51° | | 0.7 | 12.6 | | 98.07% | | 24.64% | |
| Median |  | 983 | 998 | | 198 | | 520 | 223 | | 275 | | 427 | 152 | | 602 | | 329 | 558 | | 99 | 120 | | 137 | | 29 | 255 | | 45 | | 126° | 32° | | 0.5 | 6.4 | | 95.99% | | 17.09% | |
| SD |  | 562 | 517 | | 112 | | 326 | 25 | | 25 | | 189 | 75 | | 196 | | 109 | 176 | | 53 | 25 | | 89 | | 15 | 126 | | 29 | | 8 | 9 | | 0.1 | 1.8 | | 2.03% | | 3.94% | |
| V |  | L_m_/L | | L_ms_/L | | L_ms_/W_ms_ | | | L_n_/L | | L_p_/L | | | W_p_/W | | L_p_/W_p_ | | | L_g_/L_p_ | | | W_g_/W_p_ | | L_g_/W_g_ | | | L_pl_/L | | W_pl_/W | | | L_p-i_/L_p-e_ | | | W_p-i_/W_p-e_ | | A_g_/A | |  |
| N |  | 11 | | 15 | | 19 | | | 4 | | 12 | | | 10 | | 13 | | | 14 | | | 13 | | 17 | | | 5 | | 5 | | | 12 | | | 11 | | 14 | |  |
| Mean |  | 56.67% | | 20.77% | | 81.21% | | | 36.02% | | 11.45% | | | 44.18% | | 24.43% | | | 64.22% | | | 20.38% | | 87.27% | | | 18.19% | | 38.27% | | | 55.66% | | | 60.48% | | 27.18% | |  |
| Min |  | 53.50% | | 8.60% | | 68.81% | | | 32.65% | | 7.79% | | | 35.75% | | 18.09% | | | 42.41% | | | 15.68% | | 49.41% | | | 13.77% | | 28.00% | | | 20.63% | | | 30.51% | | 18.44% | |  |
| Max |  | 62.80% | | 31.87% | | 94.84% | | | 43.20% | | 14.40% | | | 55.21% | | 30.09% | | | 83.19% | | | 26.56% | | 146.72% | | | 21.96% | | 51.60% | | | 73.01% | | | 88.89% | | 42.86% | |  |
| Median |  | 56.68% | | 22.84% | | 80.77% | | | 34.13% | | 11.31% | | | 43.01% | | 26.14% | | | 62.16% | | | 19.96% | | 79.84% | | | 17.89% | | 39.21% | | | 56.86% | | | 57.69% | | 24.07% | |  |
| SD |  | 2.69% | | 8.36% | | 7.15% | | | 4.90% | | 2.28% | | | 6.84% | | 4.04% | | | 10.66% | | | 3.35% | | 28.62% | | | 3.03% | | 8.96% | | | 15.44% | | | 18.96% | | 7.91% | |  |

| D | L | W | | H | | L_m_ | | L_ms_ | | W_ms_ | | L_r_ | L_p_ | | W_p_ | | L_u_ | | W_u_ | L_g_ | | W_g_ | A | A_g_ | Pi | Pu | L/W | H/L | L_m_/L | L_ms_/L | L_ms_/W_ms_ |
| --- | --- | --- | --- | --- | --- | --- | --- | --- | --- | --- | --- | --- | --- | --- | --- | --- | --- | --- | --- | --- | --- | --- | --- | --- | --- | --- | --- | --- | --- | --- | --- |
| N | 16 | 15 | | 16 | | 12 | | 10 | | 10 | | 9 | 11 | | 11 | | 5 | | 5 | 7 | | 5 | 13 | 4 | 17 | 41 | 15 | 16 | 12 | 10 | 10 |
| Mean | 1116 | 1240 | | 229 | | 569 | | 198 | | 260 | | 746 | 112 | | 579 | | 283 | | 734 | 104 | | 292 | 136° | 112° | 0.5 | 6.3 | 92.6% | 19.5% | 55.6% | 18.6% | 76.0% |
| Min | 624 | 669 | | 107 | | 365 | | 150 | | 227 | | 359 | 50 | | 305 | | 178 | | 544 | 61 | | 139 | 115° | 101° | 0.3 | 3.1 | 86.5% | 15.0% | 49.9% | 9.7% | 63.0% |
| Max | 2107 | 2190 | | 550 | | 1003 | | 238 | | 309 | | 1243 | 205 | | 1091 | | 431 | | 880 | 160 | | 533 | 158° | 127° | 0.7 | 11.4 | 97.8% | 26.1% | 60.7% | 29.6% | 89.1% |
| Median | 897 | 1056 | | 176 | | 498 | | 206 | | 254 | | 603 | 95 | | 502 | | 270 | | 835 | 93 | | 287 | 140° | 111° | 0.5 | 6.4 | 93.4% | 18.2% | 56.7% | 17.9% | 75.3% |
| SD | 498 | 526 | | 140 | | 198 | | 33 | | 29 | | 357 | 48 | | 265 | | 104 | | 172 | 41 | | 158 | 13 | 11 | 0.1 | 1.8 | 3.7% | 3.5% | 3.1% | 7.0% | 9.6% |
| D | L_r_/L | | L_p_/L | | W_p_/W | | L_g_/L_p_ | | L_g_/W_g_ | | W_g_/W_p_ | | | L_u_/L | | W_u_/W | | A_g_/A | | |  |  |  |  |  |  |  |  |  |  |  |
| N | 9 | | 11 | | 11 | | 7 | | 5 | | 5 | | | 5 | | 5 | | 3 | | |  |  |  |  |  |  |  |  |  |  |  |
| Mean | 52.9% | | 8.8% | | 41.0% | | 83.6% | | 34.8% | | 45.2% | | | 16.2% | | 39.4% | | 76.7% | | |  |  |  |  |  |  |  |  |  |  |  |
| Min | 32.4% | | 6.3% | | 35.6% | | 78.0% | | 27.5% | | 30.9% | | | 12.8% | | 36.9% | | 72.2% | | |  |  |  |  |  |  |  |  |  |  |  |
| Max | 61.4% | | 13.0% | | 50.4% | | 88.8% | | 44.6% | | 62.7% | | | 22.3% | | 42.1% | | 82.5% | | |  |  |  |  |  |  |  |  |  |  |  |
| Median | 53.6% | | 8.5% | | 39.9% | | 84.9% | | 36.2% | | 45.0% | | | 16.2% | | 39.3% | | 75.4% | | |  |  |  |  |  |  |  |  |  |  |  |
| SD | 9.3% | | 1.9% | | 5.0% | | 4.1% | | 7.0% | | 11.4% | | | 3.9% | | 1.9% | | 5.3% | | |  |  |  |  |  |  |  |  |  |  |  |

All measurements are in μm. Abbreviations: A, apical angle; A_g_, angle of ventral pedicle groove or dorsal median groove; D, dorsal valve; Pi, diameter of pitted structures; Pu, diameter of pustules; V, ventral valve. L, length; W, width; H, height of valve where not specified, and of elements: g, ventral pedicle groove or dorsal median groove; m, valve length at the maximum width; r, median ridge; ms, metamorphic shell; p, pseudointerarea; p-i, inner part of proparea; p-o, outer part of proparea; pl, posterolateral muscle scars; pu, pustules, n, pedicle nerve; u, umbonal muscle scars.
