# Supplementary material for: Evolution and diversity of biomineralized columnar architecture in early Cambrian phosphatic-shelled brachiopods
Source: eLife. 2024 Apr 10;12:RP88855. doi: 10.7554/eLife.88855 (PMC11006422; doi:10.7554/eLife.88855)
Supplement: Supplementary file 2. [file elife-88855-supp2.docx]

**Supplementary file 2**. Average dimensions and ratios of ventral and dorsal valves of *Eoobolus acutulus* sp. nov. from the Cambrian Series 2 Shuijingtuo Formation, South China.

| V | | L | W | | H | | L_m_ | L_ms_ | | W_ms_ | | L_n_ | L_p_ | | W_p_ | | L_pl_ | W_pl_ | | L_g_ | | W_g_ | L_p-i_ | | W_p-i_ | | L_p-e_ | W_p-e_ | | A | A_g_ | | Pi | | L_pu_ | W_pu_ | | L/W | |  |
| --- | --- | --- | --- | --- | --- | --- | --- | --- | --- | --- | --- | --- | --- | --- | --- | --- | --- | --- | --- | --- | --- | --- | --- | --- | --- | --- | --- | --- | --- | --- | --- | --- | --- | --- | --- | --- | --- | --- | --- | --- |
| N | | 13 | 10 | | 5 | | 11 | 11 | | 12 | | 1 | 9 | | 7 | | 2 | 2 | | 10 | | 12 | 11 | | 12 | | 10 | 11 | | 20 | 11 | | 68 | | 18 | 18 | | 10 | |  |
| Mean | | 1211 | 979 | | 285 | | 756 | 229 | | 255 | | 539 | 594 | | 1079 | | 459 | 673 | | 341 | | 251 | 443 | | 155 | | 1066 | 137 | | 83° | 17° | | 0.6 | | 11.8 | 4.7 | | 129.53% | |  |
| Min | | 715 | 562 | | 178 | | 442 | 181 | | 191 | | 539 | 178 | | 509 | | 329 | 548 | | 102 | | 141 | 128 | | 30 | | 348 | 43 | | 73° | 6° | | 0.2 | | 8.0 | 2.7 | | 122.90% | |  |
| Max | | 1877 | 1466 | | 432 | | 1110 | 277 | | 301 | | 539 | 1096 | | 1877 | | 589 | 798 | | 629 | | 567 | 1027 | | 406 | | 2933 | 312 | | 89° | 31° | | 1.1 | | 21.0 | 7.2 | | 136.16% | |  |
| Median | | 1170 | 992 | | 295 | | 784 | 237 | | 263 | | 539 | 487 | | 974 | | 459 | 673 | | 366 | | 211 | 382 | | 150 | | 908 | 135 | | 84° | 17° | | 0.6 | | 10.9 | 4.8 | | 130.36% | |  |
| SD | | 335 | 280 | | 96 | | 210 | 37 | | 30 | | 0 | 328 | | 463 | | 184 | 177 | | 187 | | 117 | 275 | | 105 | | 763 | 78 | | 5 | 9 | | 0.2 | | 3.4 | 1.3 | | 4.80% | |  |
| V | H/L | | | L_m_/L | | L_ms_/L | | | L_ms_/W_ms_ | | L_n_/L | | | L_p_/L | | W_p_/W | | | L_p_/W_p_ | | L_g_/L_p_ | | | W_g_/W_p_ | | L_g_/W_g_ | | | L_pl_/L | | | W_pl_/W | | L_p-i_/L_p-e_ | | | W_p-i_/W_p-e_ | | A_g_/A | |
| N | 4 | | | 11 | | 8 | | | 10 | | 1 | | | 4 | | 3 | | | 6 | | 5 | | | 6 | | 8 | | | 2 | | | 2 | | 9 | | | 10 | | 10 | |
| Mean | 21.49% | | | 60.79% | | 19.53% | | | 92.06% | | 46.03% | | | 24.73% | | 68.79% | | | 53.52% | | 43.40% | | | 20.12% | | 155.51% | | | 31.40% | | | 62.94% | | 40.92% | | | 103.65% | | 21.54% | |
| Min | 16.05% | | | 55.85% | | 10.07% | | | 73.83% | | 46.03% | | | 19.16% | | 56.56% | | | 47.59% | | 38.81% | | | 10.28% | | 69.01% | | | 28.10% | | | 62.15% | | 32.81% | | | 69.77% | | 6.74% | |
| Max | 25.46% | | | 67.01% | | 25.31% | | | 109.91% | | 46.03% | | | 28.70% | | 75.86% | | | 59.94% | | 57.30% | | | 31.04% | | 229.02% | | | 34.71% | | | 63.72% | | 56.03% | | | 156.21% | | 35.62% | |
| Median | 22.23% | | | 61.34% | | 20.51% | | | 95.51% | | 46.03% | | | 25.53% | | 73.95% | | | 53.68% | | 40.33% | | | 19.02% | | 155.29% | | | 31.40% | | | 62.94% | | 39.80% | | | 93.02% | | 21.94% | |
| SD | 4.62% | | | 3.01% | | 4.87% | | | 11.80% | | #DIV/0! | | | 4.02% | | 10.64% | | | 5.66% | | 7.87% | | | 7.99% | | 57.93% | | | 4.68% | | | 1.11% | | 7.54% | | | 30.35% | | 10.87% | |

| D | L | W | L_m_ | L_p_ | W_p_ | L_g_ | A | L/W | L_m_/L | L_p_/L | W_p_/W | L_g_/L_p_ |
| --- | --- | --- | --- | --- | --- | --- | --- | --- | --- | --- | --- | --- |
| N | 2 | 2 | 2 | 2 | 2 | 2 | 2 | 2 | 2 | 2 | 2 | 2 |
| Mean | 1203 | 939 | 723 | 229 | 637 | 165 | 90° | 127.7% | 59.7% | 18.8% | 67.4% | 74.7% |
| Min | 1015 | 817 | 578 | 178 | 519 | 156 | 87° | 124.2% | 56.9% | 17.5% | 63.5% | 61.8% |
| Max | 1390 | 1060 | 867 | 280 | 755 | 173 | 93° | 131.1% | 62.4% | 20.1% | 71.2% | 87.6% |
| Median | 1203 | 939 | 723 | 229 | 637 | 165 | 90° | 127.7% | 59.7% | 18.8% | 67.4% | 74.7% |
| SD | 265 | 172 | 204 | 72 | 167 | 12 | 4 | 4.9% | 3.8% | 1.8% | 5.4% | 18.3% |

All measurements are in μm. Abbreviations: A, apical angle; A_g_, angle of ventral pedicle groove or dorsal median groove; D, dorsal valve; Pi, diameter of pitted structures; V, ventral valve. L, length; W, width; H, height of valve where not specified, and of elements: g, ventral pedicle groove or dorsal median groove; m, valve length at the maximum width; r, median ridge; ms, metamorphic shell; p, pseudointerarea; p-i, inner part of proparea; p-o, outer part of proparea; pl, posterolateral muscle scars; pu, pustules, n, pedicle nerve; u, umbonal muscle scars.
